# Oxidative Stress and Low-Grade Endotoxemia in Pediatric Acute-Onset Neuropsychiatric Syndrome (PANS) and Paediatric Autoimmune Neuropsychiatric Disorders Associated with Streptococcal Infections (PANDAS): Insights from a Cross-Sectional Study

**DOI:** 10.3390/ijms26136336

**Published:** 2025-07-01

**Authors:** Chiara Maria Totè, Martina Capponi, Francesca Salvatori, Anna Maria Zicari, Cristiana Alessia Guido, Giulia Brindisi, Laura Iantorno, Simona Bartimoccia, Francesco Baratta, Maurizio Forte, Vittorio Picchio, Mariaelena Malvasi, Simone Aloisio, Elena Pacella, Pasquale Pignatelli, Francesco Violi, Roberto Carnevale, Alberto Spalice, Lorenzo Loffredo

**Affiliations:** 1 Department of Clinical Internal, Anaesthetic and Cardiovascular Science, Sapienza University of Rome, Viale del Policlinico, 155, 00161 Rome, RM, Italy; chiaramaria.tote@uniroma1.it (C.M.T.); iantorno.laura@yahoo.it (L.I.); francesco.baratta@uniroma1.it (F.B.); pasquale.pignatelli@uniroma1.it (P.P.); francesco.violi@uniroma1.it (F.V.); 2 Department of Mother-Child, Urological Science, La Sapienza University, 00161 Rome, RM, Italy; m.capponi@uniroma1.it (M.C.); f.salvatori@uniroma1.it (F.S.); annamaria.zicari@uniroma1.it (A.M.Z.); cristiana.guido@uniroma1.it (C.A.G.); giulia.brindisi@uniroma1.it (G.B.); 3 Department of Medical-Surgical Sciences and Biotechnologies, Sapienza University of Rome, 04100 Latina, LT, Italy; simona.bartimoccia@uniroma1.it (S.B.); roberto.carnevale@uniroma1.it (R.C.); 4 IRCCS Neuromed, 86077 Pozzilli, IS, Italy; maurizio.forte@neuromed.it (M.F.); vittorio.picchio@uniroma1.it (V.P.); 5 Department of Sense Organs, Sapienza University of Rome, 00185 Rome, RM, Italy; mariaelena.malvasi@uniroma1.it (M.M.); elena.pacella@uniroma1.it (E.P.); 6 Department of Human Neuroscience, Sapienza University of Rome, Viale dell’Università 30, 00185 Rome, RM, Italy; aloisiosimone10@gmail.com

**Keywords:** PANS, PANDAS, NOX2, NADPH oxidase, oxidative stress, LPS

## Abstract

Pediatric Autoimmune Neuropsychiatric Disorders Associated with Streptococcal Infections (PANDAS), unlike pediatric acute-onset neuropsychiatric syndrome (PANS), is triggered by infections. This study aimed to assess the differences in low-grade endotoxemia and oxidative stress between these conditions. A cross-sectional study compared serum levels of soluble NOX2-dp (sNOX-2-dp), isoprostanes, lipopolysaccharide (LPS), and zonulin in 30 PANDAS, 21 PANS, and 30 control (CT) children matched for age and gender. Zonulin was used to assess gut permeability. Patients with PANDAS showed significantly higher serum levels of sNOX2-dp, isoprostanes, LPS, and zonulin than PANS and controls, while no significant differences were found between PANS and controls. sNOX2-dp correlated with isoprostanes (Rs = 0.708; *p* < 0.001), LPS (Rs = 0.584; *p* < 0.001), and zonulin (Rs = 0.662; *p* < 0.001). Multiple regression identified isoprostanes (β = 0.599; *p* < 0.001) and zonulin (β = 0.295; *p* = 0.01) as independent predictors of sNOX2-dp (R^2^ = 81%). PANDAS and PANS showed distinct profiles of LPS, zonulin, NOX2, and isoprostanes. Future research should explore therapies targeting endotoxemia and oxidative stress for potential clinical benefits.

## 1. Introduction

Neurological disorders in children are frequently linked to infections, which can harm the nervous system either directly or by initiating immune-related pathways [1]. Among the infectious agents, *Streptococcus pyogenes*—a Group A β-hemolytic strain—is known to provoke immune responses that may adversely affect the central nervous system (CNS) [2,3,4].

Post-infectious neurological complications associated with *S. pyogenes* can manifest as various movement abnormalities—such as chorea, tics, dystonia, and parkinsonian features—as well as psychiatric symptoms [2,5]. These clinical manifestations are encompassed under the term Pediatric Autoimmune Neuropsychiatric Disorders Associated with Streptococcal Infections (PANDAS), a condition within a wider clinical framework that includes tic disorders and obsessive-compulsive behaviors, the Pediatric Acute-onset Neuropsychiatric Syndrome (PANS) [6].

Emerging evidence suggests that imbalances in gut microbiota—commonly referred to as intestinal dysbiosis—may play a role in both cardiovascular [7] and neurological diseases [8]. This has led to growing interest in the gut–brain axis, a bidirectional communication system between the gastrointestinal tract and the nervous system [8,9]. Once in circulation, LPS can engage Toll-like receptor 4 (TLR4), setting off inflammatory cascades and increasing oxidative stress [10]. A key player in this process is NADPH oxidase 2 (NOX2), an enzyme that facilitates the production of reactive oxygen species, thereby exacerbating systemic and neural inflammation [11,12].

Recent investigations have increasingly focused on the role of gut dysbiosis in the pathophysiology of PANS and PANDAS. Research by Quagliarello et al. revealed that children diagnosed with these conditions display distinct gut microbial profiles when compared to healthy individuals, particularly with increased levels of *Bacteroides*, *Odoribacter*, and *Oscillospira*, genera that may contribute to inflammation and autoimmunity [13]. Furthermore, our own research has demonstrated that children with PANDAS exhibit elevated blood levels of LPS, NOX2, and isoprostanes, indicating heightened oxidative stress relative to control subjects [14].

Since PANDAS is characterized by its association with streptococcal infection, this study aimed to determine whether there are measurable differences in low-grade endotoxemia and oxidative stress markers between patients with PANDAS and those with PANS.

## 2. Results

Eighty-one consecutive children were enrolled in the study. The clinical characteristics of patients with PANS, PANDAS, and controls are presented in Table 1. No significant differences were observed among the three groups in terms of age, fasting blood glucose, BMI, or systolic and diastolic blood pressure (Table 1). Furthermore, no significant differences were found between the PANS and PANDAS groups regarding the presence of OCD or tic disorders. As expected, patients with PANDAS showed higher anti-streptolysin O (ASO) titers compared to those with PANS.

**Figure 1:**
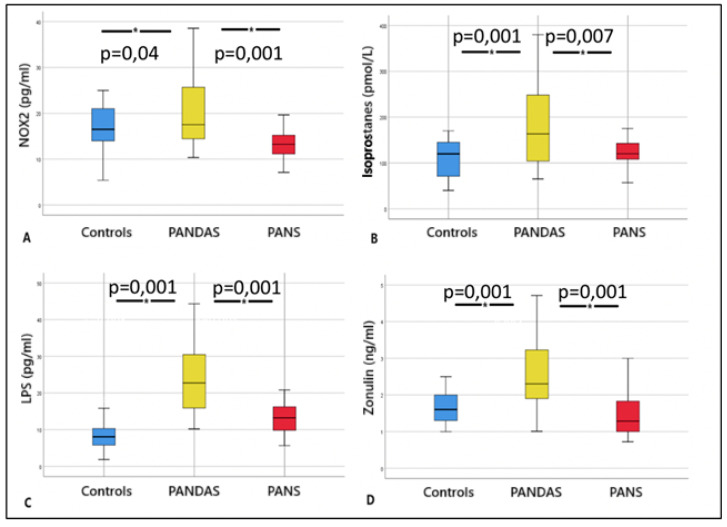
Box whisker plots of sNOX2-dp (**A**), serum isoprostanes (**B**), LPS (**C**), and zonulin (**D**) in controls, PANDAS and PANS. The controls are shown in blue, the PANDAS in yellow, and the PANS in red. * The asterisks indicate a comparison with the significant *p*.

Serum levels of sNOX2-dp, isoprostanes, LPS, and zonulin were significantly higher in patients with PANDAS compared to both PANS patients and healthy controls (Table 1 and Figure 1A–D). No significant differences were observed between PANS patients and controls for any of the markers (sNOX2-dp, isoprostanes, LPS, or zonulin).

**Figure 2:**
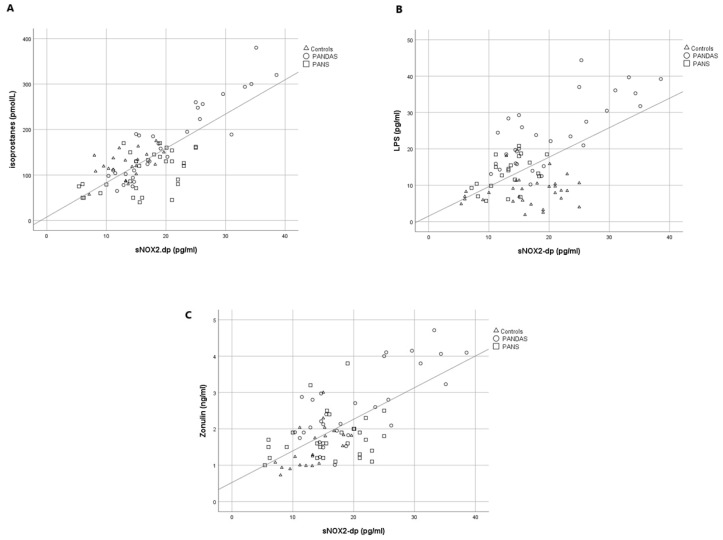
Linear correlation analysis between levels of sNOX2-dp with serum isoprostanes (**A**), LPS (**B**), and zonulin (**C**).

Simple linear regression analysis, conducted across all participants, revealed that sNOX2-dp was significantly correlated with serum isoprostanes (Rs = 0.708; *p* < 0.001, Figure 2A), LPS (Rs = 0.584; *p* < 0.001, Figure 2B), and zonulin (Rs = 0.662; *p* < 0.001, Figure 2C).

Additionally, LPS was significantly correlated with serum isoprostanes (Rs = 0.708; *p* < 0.001) and zonulin (Rs = 0.729; *p* < 0.001). Moreover, isoprostanes were correlated with tic disorders (Rs = 0.382; *p* = 0.03).

Multiple linear regression analyses, including variables that were linearly associated with the dependent variable, such as isoprostanes, LPS, and zonulin, added to age and gender, were performed to identify independent predictors of sNOX2-dp in the overall population. Isoprostanes (SE: 0.012; standardized coefficient β: 0.599; *p* < 0.001) and zonulin (SE: 0.634; standardized coefficient β: 0.295; *p* = 0.01) emerged as the only independent predictors of sNOX2-dp (R^2^ = 81%).

## 3. Discussion

This study highlights distinct differences in low-grade endotoxemia and oxidative stress markers between PANS and PANDAS. The results indicate that PANDAS should not be simply viewed as a subset of PANS; instead, these disorders might share overlapping clinical manifestations while having distinct pathophysiological mechanisms.

A particularly notable finding concerns the divergent levels of circulating lipopolysaccharide (LPS) and zonulin between children with PANS and those with PANDAS. Elevated levels of endotoxins and zonulin have been previously associated with a range of neurological diseases, including Alzheimer’s, Parkinson’s, and amyotrophic lateral sclerosis (ALS) [9,15]. These findings lend further support to the “gut–brain axis” hypothesis, suggesting that gastrointestinal permeability and microbial products may influence neuroinflammation, thus playing a role in disease development [15]. In animal models, circulating LPS has been shown to provoke neuroinflammatory responses by enhancing interleukin-1 beta expression, which disrupts synaptic function and neuronal signaling—as confirmed by changes in EEG patterns [16]. More recently, elevated LPS levels have also been implicated in pediatric neurodevelopmental disorders, including autism spectrum disorders [17,18,19,20]. One study using an autism mouse model found that increased gut permeability enabled LPS to reach the brain, triggering the TLR4/MyD88/NF-κB pathway and creating a pro-inflammatory environment [21].

Additionally, emerging evidence suggests a distinct gut microbial profile in patients with PANS/PANDAS, characterized by a greater abundance of genera such as *Bacteroides*, *Odoribacter*, and *Oscillospira* [13]. Quagliariello et al. explored the complex bidirectional communication between the gut microbiome and the central nervous system in PANDAS, documenting significant disruptions in the intestinal microbial ecosystem [13]. These microbial imbalances may contribute indirectly to brain inflammation and diminish the availability of vital neuroactive metabolites such as short-chain fatty acids (SCFAs), as well as affect key pathways like D-alanine metabolism, tyrosine metabolism, and dopamine synthesis [13]. However, their study did not distinguish between patients with PANS and those with PANDAS—highlighting a potential gap, as differences in gut microbiota composition between the two disorders remain underexplored.

LPS, via activation of the TLR4 receptor, stimulates NOX2, resulting in increased oxidative stress [10]. Therefore, a greater passage of LPS into the bloodstream could exacerbate inflammation and oxidative damage, potentially contributing to the onset or severity of PANDAS [14]. This pathway may be influenced by gut microbial changes following *Streptococcus pyogenes* infections, as has been noted in the nasal and vaginal microbiomes [22,23].

**Figure 3:**
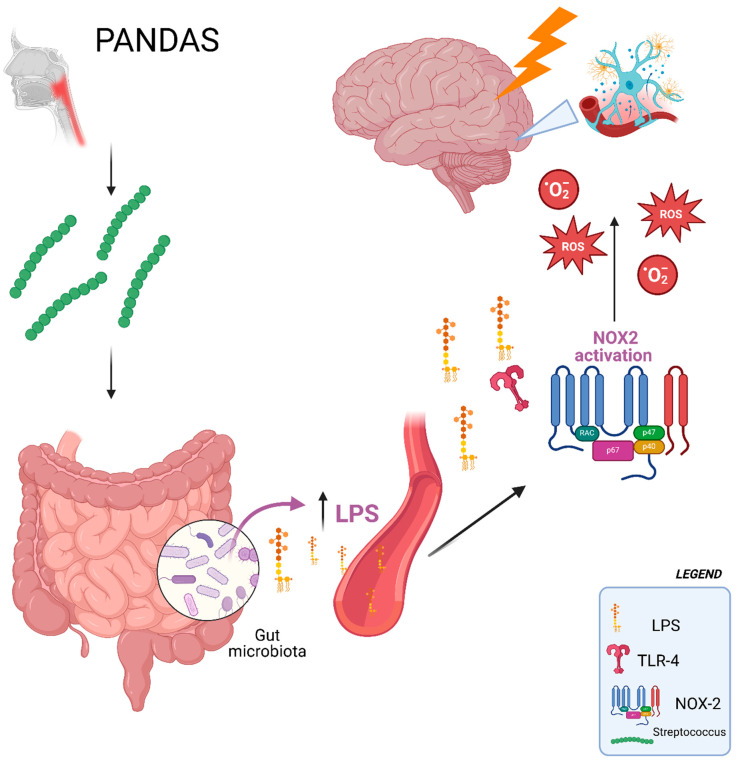
In children with PANDAS, unlike those with PANS, the increase in low levels of endotoxemia could, through binding to TLR4, activate NOX2. This, in turn, might lead to increased oxidative stress at both the systemic and cerebral levels, promoting neuroinflammation.

Given the higher concentrations of zonulin, sNOX2-dp, isoprostanes, and LPS in patients with PANDAS relative to those with PANS [14], it can be speculated that PANDAS involves a specific dysregulation of the gut microbiota, contributing to neuroinflammation through LPS-mediated NOX2 activation and increased oxidative stress (see Figure 3).

### 3.1. Limitations

This study has some limitations. First, it does not directly assess gut microbiota composition, preventing a definitive link between LPS elevation and specific microbial populations. Second, oxidative stress and NOX2 activity were measured in peripheral circulation, not in brain tissue. Third, the limited sample size necessitates larger-scale studies to confirm these preliminary findings. Fourth, another limitation of the study is the absence of measurements for molecules such as butyrate, which play a key role in regulating intestinal homeostasis by maintaining gut barrier integrity [24]. Furthermore, a methodological limitation of this study is that the ELISA kit used to quantify zonulin may not specifically detect zonulin itself. Instead, it might cross-react with structurally related proteins, such as properdin, as previously demonstrated by Scheffler et al. [25]. This cross-reactivity could potentially compromise the assay’s specificity.

### 3.2. Clinical Implications

Despite these limitations, the findings may hold clinical relevance.

Distinguishing between PANS and PANDAS may aid in identifying patients who could benefit from personalized therapeutic approaches—such as probiotics like Escherichia coli Nissle 1917, which has shown efficacy in neurodegenerative diseases like Alzheimer’s disease by reducing levels of LPS, zonulin, and NOX2 [26], dietary interventions [27], or antioxidant treatments targeting NOX2 activation and oxidative stress.

## 4. Methods

From January 2018 to September 2023, a total of 81 consecutive patients referred to the Departments of Allergology and Pediatric Neurology at the Sapienza University of Rome were recruited for this investigation. Additionally, a control group composed of 30 age- and sex-matched individuals (24 boys and 6 girls, average age 9 ± 3 years) was enrolled during the same timeframe from the same institution, through a pediatric health screening initiative.

Eligible participants were children between the ages of 3 and 16 who had been diagnosed with Pediatric Autoimmune Neuropsychiatric Disorders Associated with Streptococcal infections, as defined by the diagnostic framework developed by Dr. Susan Swedo [28,29] and the guidelines developed by the PANS/PANDAS Research Consortium [30], which includes the following criteria:

- Clinical diagnosis of Obsessive–Compulsive Disorder (OCD) and/or tic disorders.
- Symptom onset occurring between the age of 3 and the onset of puberty.
- Disease presenting in a relapsing–remitting pattern.
- Symptom exacerbations temporally related to group A streptococcal (GAS) infections.
- Presence of neurological features such as choreiform movements or motor hyperactivity during flare-ups.

Exclusion criteria comprised:

- Sydenham’s chorea
- Tourette syndrome
- Autoimmune encephalitis
- Systemic autoimmune diseases
- Wilson’s disease
- Congenital cardiac anomalies
- Chronic kidney disease
- Malignancies
- Hepatic failure
- Acute illnesses
- Any prior treatment with immunosuppressants
- Diabetes
- Dyslipidemia
- Hypertension
- Use of antibiotics, probiotics, or antioxidant therapies within the 4 weeks prior to enrollment in the study.

### 4.1. Blood Collection

Blood samples were drawn between 8:00 and 9:00 AM following a 12 h overnight fast. Specimens were collected using Vacutainer tubes (Vacutainer Systems, Belliver Industrial Estate, Plymouth, UK), centrifuged at 300× *g* for 10 min, and the supernatant was stored at −80 °C for subsequent biochemical and oxidative stress analyses.

### 4.2. Measurement of sNOX2-Dp

The serum concentration of soluble NOX2-derived peptide (sNOX2-dp) was quantified using an enzyme-linked immunosorbent assay (ELISA), following a previously described protocol [31]. ELISA plates were coated overnight at 4 °C with either reference standards (0–200 pg/mL) or platelet supernatants (1 µg protein). After blocking non-specific sites for 2 h at room temperature, wells were incubated with a horseradish peroxidase (HRP)-conjugated monoclonal antibody specific to the extracellular domain of NOX2. Detection was performed using 3,3′,5,5′-tetramethylbenzidine (TMB) substrate, and absorbance was read at 450 nm post-reaction termination with 2 M sulfuric acid. The sNOX2-dp concentration was extrapolated from a standard curve and reported in pg/mL. The intra-assay and inter-assay variability were 8.95% and 9.01%, respectively.

### 4.3. Quantification of 8-Iso-Prostaglandin F2α

Serum levels of 8-iso-Prostaglandin F2α (8-iso-PGF2α) were quantified using commercial competitive enzyme-linked immunosorbent assay (ELISA) kits from Abcam (Cambridge, UK) and DRG International, Inc. (Springfield, NJ, USA). Serum samples and standards were added to microplate wells pre-coated with anti-8-iso-PGF2α antibodies, followed by incubation with an enzyme-conjugated tracer. After washing to remove unbound components, a colorimetric substrate was added. The enzymatic reaction produced a color change inversely proportional to the concentration of 8-iso-PGF2α in the samples. Absorbance was measured at 450 nm (Abcam) or 405 nm (DRG) using a microplate reader.

### 4.4. Zonulin Detection

Serum zonulin concentrations were measured by ELISA (Elabscience). Plates pre-coated with anti-zonulin antibodies were incubated with 100 μL of standard or sample for 90 min at 37 °C. Detection was completed using a biotinylated anti-zonulin antibody followed by avidin-HRP. Signal intensity was read at 450 nm using an automated microplate reader. Results were expressed in ng/mL, with intra- and inter-assay coefficients of variation both below 10%.

### 4.5. Lipopolysaccharide (LPS) Analysis

Frozen plasma samples were thawed once and subjected to a sandwich ELISA for LPS quantification (Hycult Biotechnology, Uden, The Netherlands), with a detection range between 0.04 and 10.0 EU/mL.

### 4.6. Statistical Analysis

Statistical analyses were performed using SPSS software version 18.0 for Windows (SPSS Inc., Chicago, IL, USA). The Shapiro–Wilk test was used to assess the normality of variable distributions. Data with a normal distribution are presented as means ± standard deviations (SD). Group differences were evaluated using the Kruskal–Wallis test for non-normally distributed data or analysis of variance (ANOVA) for normally distributed data. Differences in proportions were assessed using the chi-square (χ^2^) test. Bivariate correlations were examined using the Spearman’s rank correlation. Variables with a *p*-value < 0.10 in bivariate analysis were included in a multivariable linear regression model using an automated forward selection procedure. A *p*-value < 0.05 was considered statistically significant.

A multivariate regression analysis was conducted including two predictors. According to Green’s rule of thumb [32], a minimum sample size of 66 subjects is required to detect medium effect sizes when testing the overall model (N ≥ 50 + 8 × m, where m = number of predictors). Given our sample size of 81 subjects, the analysis meets this recommended criterion.

## 5. Conclusions

In summary, this study reveals that PANS and PANDAS are associated with distinct serum levels of LPS, zonulin, NOX2, and isoprostanes. Further research is warranted to investigate the efficacy of therapeutic approaches targeting endotoxemia and oxidative stress, with the goal of improving clinical outcomes in affected pediatric populations.

## Figures and Tables

**Table 1:** Clinical characteristics of controls and patients. *p* < 0.05 compared to PANDAS.

	PANDAS (n = 30)	PANS (n = 21)	Controls (n = 30)
**Age**	9 ± 3	9 ± 3	9 ± 3
**Gender (male/female)**	24/6	15/6	24/6
**Glycaemia (mg/dL)**	83 ± 3.75	85 ± 3.6	87 ± 3
**Systolic blood pressure (mmHg)**	110 ± 3	109 ± 3	112 ± 3
**Diastolic blood pressure (mmHg)**	67 ± 2.55	68 ± 2	70 ± 2
**BMI**	18 ± 2	18 ± 2	17 ± 1
**Tic disorders (presence/absence)**	25/5	19/2	0
**OCD (presence/absence)**	10/20	11/10	0
**Anti-streptolysinic titer (UI/mL)**	409 ± 262	264 ± 21°	0
**LPS (pg/mL)**	24.1 ± 9.2	13.1 ± 4.5°	8.1 ± 3.6°
**NOX2 (pg/mL)**	20.4 ± 8.1	13.2 ± 4.8°	16.3 ± 5.7°
**Zonulin (ng/mL)**	2.6 ± 1	1.5 ± 0.6°	1.7 ± 0.6°
**Isoprostanes (pmol/L)**	175 ± 84	122 ± 30°	106 ± 43°

## Data Availability

Data are available on request to the corresponding author.
